# Lymph Node Dissection and Postoperative Complications After Lung Cancer Resection

**DOI:** 10.1001/jamanetworkopen.2026.15894

**Published:** 2026-06-01

**Authors:** Isheeta Madeka, Khaled Noueihed, Jacob Woodroof, Shale Mack, Hamza Rshaidat, Gregory L. Whitehorn, Sneha Alaparthi, Scott H. Koeneman, Tyler R. Grenda, Nathaniel R. Evans, Olugbenga T. Okusanya

**Affiliations:** 1 Department of Surgery, Division of Esophageal and Thoracic Surgery, Thomas Jefferson University Hospital, Philadelphia, Pennsylvania; 2 Department of Surgery, University of Chicago Hospital, Chicago, Illinois; 3 Department of Surgery, University of Maryland Medical Center, Baltimore; 4 Division of Biostatistics and Bioinformatics, Thomas Jefferson University, Philadelphia, Pennsylvania

## Abstract

**Importance:**

Current lung cancer guidelines recommend lymph node sampling (LNS) with the 3 + 1 rule, which recommends station-based sampling with at least 3 N2 (mediastinal) and 1 N1 (hilar) nodal stations. Although there is oncologic benefit to rigorous LNS, potential negative impacts on surgical outcomes have not been well investigated. As adoption of the 3 + 1 rule increases given its formalization as guideline-concordant care, associations with adverse outcomes are crucial to characterize.

**Objective:**

To evaluate whether satisfying the 3 + 1 rule is associated with increased postoperative complications.

**Design, Setting, and Participants:**

This retrospective cohort study used the Society of Thoracic Surgeons General Thoracic Database, which was queried for patients with clinical stage T1 to T3, N0, M0 non–small cell lung cancer (NSCLC) who underwent surgical resection with known LNS between July 1, 2021, and January 1, 2023. Statistical analysis was performed from March 2024 to March 2025.

**Exposure:**

A propensity match was conducted to compare the postoperative complication rate by 3 + 1 LNS status.

**Main Outcomes and Measures:**

Data on demographic characteristics were collected and analyzed. Primary outcomes included postoperative complications, such as arrhythmias, pleural effusion requiring drainage, blood transfusion, prolonged air leak, therapeutic bronchoscopy, pneumonia, respiratory failure, bronchopleural fistula, pulmonary embolism, pneumothorax, prolonged ventilatory support, myocardial infarction, and sepsis. Thirty-day mortality and readmission rates were also evaluated.

**Results:**

The cohort included 28 439 patients (median [IQR] age, 69 [66-75] years; 4791 [59.5%] female). There were 18 939 patients (66.6%) who satisfied the 3 + 1 rule. In the unadjusted group, the 3 + 1 cohort had longer median (IQR) operating room duration (224 [178-281] vs 210 [161-273] minutes, *P* < .001) and rates of pathologic upstaging (2520 [13.3%] vs 922 [9.7%], *P* < .001). In the postmatch analysis, there were no other significant differences in postoperative complication rates, and the aforementioned associations in the 3 + 1 group were no longer observed.

**Conclusions and Relevance:**

In this cohort study of patients with NSCLC, the 3 + 1 rule was not associated with increased postoperative complications. This study’s findings suggest support for the continued use of this strategy in terms of overall safety profile.

## Key Points

**Question:**

Is lymph node sampling of at least 3 N2 (mediastinal) and 1 N1 (hilar) nodal stations (3 + 1 rule) associated with more postoperative complications?

**Findings:**

In this cohort study of 28 439 patients, the 3 + 1 rule was not associated with an increased incidence of postoperative complications in a matched cohort of patients with early-stage non–small cell lung cancer who underwent lung resection.

**Meaning:**

This study’s findings support the continued use of the 3 + 1 rule in terms of overall safety profile in concordance with current recommendations from the National Comprehensive Cancer Network and American College of Surgeons Commission on Cancer.

## Teaser

This cohort study examines whether the 3 + 1 rule (sampling of at least 3 mediastinal and 1 hilar nodal stations) is associated with a higher rate of postoperative complications among patients with non–small cell lung cancer.

## Introduction

Lung cancer is the second most common cancer in the US and remains a leading cause of cancer-related mortality. Non–small cell lung cancer (NSCLC) accounts for 80% to 85% of all lung cancers.^1,2^ Surgical resection with lymph node sampling (LNS) is standard of care for patients with early-stage NSCLC. Accurate lymph node staging is crucial for prognosis and eligibility for treatment with new agents. However, what defines adequate LNS has evolved.

Previously, mediastinal lymph node dissection (MLND) was considered standard of care. However, the ACOSOG-Z0030 randomized clinical trial (RCT) showed no difference in long-term survival or patterns of recurrence between MLND and mediastinal lymph node sampling (MLNS) in clinical T1 or T2, N0, or non-hilar N1 NSCLC.^3^ After completion of this RCT, the American College of Surgeons Commission on Cancer (ACS-CoC) recommended minimum sampling of 10 lymph nodes (LNs). However, further research showed a negligible difference in median overall survival between patients who had greater than 10 LNs sampled and less than or equal to 10 LNs.^4^ Other studies also showed variability between the number of LNs sampled and survival.^5^ Currently, the National Comprehensive Cancer Network (NCCN) guidelines and ACS-CoC recommend station-based sampling strategy, which entails sampling at least 3 N2 (mediastinal) and 1 N1 (hilar) nodal stations intraoperatively, colloquially referred to as the 3 + 1 rule.^6,7^

Although several studies^8,9,10^ have evaluated postoperative complications between MLND and MLNS, limited literature examines surgical outcomes among patients who undergo 3 + 1 LNS since its recent uptake by the NCCN and ACS-CoC. We used a national database to evaluate whether satisfying the 3 + 1 rule is associated with increased postoperative complications.

## Methods

This retrospective cohort study was conducted using the Society of Thoracic Surgeons (STS) General Thoracic Database (GTSD). The STS GTSD is the largest clinical thoracic surgical database in North America, containing approximately 800 000 general thoracic surgery procedure records with more than 900 participating surgeons. The study was deemed exempt from institutional review board approval and informed consent by Thomas Jefferson University because it is a retrospective database study. This study follows the Strengthening the Reporting of Observational Studies in Epidemiology (STROBE) reporting guideline for cohort studies.

Patients with clinical stage T1 to T3, N0, M0 NSCLC who underwent surgical resection with known LNS between July 1, 2021, and January 1, 2023, were included. We compared characteristics between patients who satisfied the 3 + 1 criteria vs those who did not. The following demographic characteristics were analyzed: age, gender, race and ethnicity, insurance, receipt of pulmonary function test, smoking history, history of cardiopulmonary disease, and history of vascular disease. Race and ethnicity data were identified from the data provided by the STS GTSD database and were collected to evaluate whether compliance with 3 + 1 lymph node sampling status differed according to patient race and ethnicity. Race and ethnicity categories included Asian, Black, Hispanic, White, and other (no further description in the STS Data Dictionary). Clinicopathological covariates of interest included clinical stage, surgical procedure, American Society of Anesthesiologists (ASA) status, Eastern Cooperative Oncology Group (ECOG) performance score, and anatomical location.

The primary outcome of interest was postoperative complications, which included overall postoperative events, atrial arrythmia, pleural effusion requiring drainage, postoperative blood transfusion, air leak (defined as any air leak lasting >5 days from operating room exit of index operation to discharge or 30 days postoperatively, whichever is longer), therapeutic bronchoscopy (the STS GTSD calls any therapeutic bronchoscopy performed within 30 days of surgery a postoperative complication), pneumonia, respiratory failure, bronchopleural fistula, pulmonary embolism, pneumothorax, ventricular arrythmia, ventilatory support for greater than 48 hours, myocardial infarction, and sepsis. Thirty-day mortality and 30-day readmission were also analyzed.

Distributions of demographic and clinicopathological characteristics and whether 3 + 1 LNS criteria were met were examined in the overall study population. Categorical variables were compared using χ^2^ tests. Continuous variables were compared using 2-tailed, unpaired *t* tests. A 1:1 propensity score match was conducted to compare postoperative complication rates by 3 + 1 LNS status. The following patient characteristics were included in the propensity match: age, gender, race and ethnicity, insurance status, receipt of pulmonary function tests, history of cardiopulmonary disease, history of vascular disease, history of smoking, clinical T stage, surgical procedure, anatomical location, robotic approach, ASA score, and ECOG score. Of note, duration of operation and length of stay (LOS) were not used in the propensity match. A caliper of 0.0000001 was used to ensure comparable postmatch cohorts. Given the multiple comparisons, an adjusted 2-sided *P* < .002 was considered statistically significant per Bonferroni correction. All analyses were conducted using Stata SE, version 17.0 (StataCorp) from March 2024 to March 2025.

## Results

Between 2021 and 2023, we identified 28 439 patients (median [IQR] age, 69 [66-75] years; 4791 [59.5%] female and 3267 [40.5%] male; 54 [0.7%] Asian, 310 [3.8%] Black, 44 [0.5%] Hispanic, 7584 [94.1%] White, and 66 [0.8%] other) with clinical stage T1 to T3, N0, M0 NSCLC who underwent surgical resection with known LNS. Of these patients, 18 939 (66.6%) satisfied the 3 + 1 rule. We compared characteristics between patients who satisfied the 3 + 1 criteria and those who did not (eTable 1 in Supplement 1). Both adjusted and unadjusted cohorts were largely similar with regard to demographic characteristics, such as age, gender, and race and ethnicity, with the exception of a few meaningful clinical characteristics. Patients in the 3 + 1 cohort were more likely to receive preoperative pulmonary function tests (18 210 [96.2%] vs 9020 [94.9%]), have T2 (3322 [17.5%] vs 1344 [14.1%]) and T3 NSCLC (1012 [5.3%] vs 390 [4.1%]), and have left-sided tumors (left upper lobe: 4892 [26.4%] vs 2365 [25.3%]; left lower lobe: 2862 [15.5%] vs 1399 [15.0%]). Patients in the 3 + 1 cohort were slightly more likely to undergo segmentectomy and pneumonectomy, with the largest difference in lobectomy (16 392 [86.6%] vs 5356 [56.3%]). They were also more likely to receive robotic surgery (12 134 [64.1%] vs 4270 [45.0%]) and have an ECOG performance score of 0 (11 306 [69.0%] vs 5300 [67.7%]). There was no difference in insurance status, smoking history, cardiovascular comorbidity, ASA score, or median (IQR) LOS (3 [2-5] vs 3 [2-5] days).

In the unadjusted group, patients who satisfied the 3 + 1 rule did not have an association with postoperative complications. However, they were more likely to have a longer median (IQR) duration of operation (224 [178-281] vs 210 [161-273] minutes, *P* < .001) and be pathologically upstaged both overall (2520 [13.3%] vs 922 [9.7%], *P* < .001) and when disaggregated by surgical approach (eTable 2 in Supplement 1). The 1:1 propensity score match yielded matched pairs of 4029 patients who satisfied the 3 + 1 rule and 4029 patients who did not (Table 1). In the postmatch analysis, there were no significant differences in overall postoperative events between the patients who satisfied the 3 + 1 rule and those who did not (1188 [29.5%] vs 1208 [30.0%], *P* = .33). Although there was a significant increase in duration of operation in the 3 + 1 unadjusted cohort, there was no significant difference in duration of operation after matching. There was a statistically significant decrease in mean (SD) LOS in the 3 + 1 postmatch cohort (4.0 [6.6] vs 4.7 [14.8] days, *P* < .001) (Table 2). Median (IQR) LOS was identical in both groups (3 [2-5] days). There was no significant difference in 30-day readmission between the patients who satisfied the 3 + 1 rule and those who did not (285 [7.1%] vs 294 [7.4%], *P* = .77). The previously observed significant increase in pathologic upstaging in the 3 + 1 cohort overall and by surgical approach was no longer observed.

**Table 1.** Propensity Score–Adjusted Demographic Characteristics

Characteristic	No. (%) of patients^a^		
	Total (N = 8058)	3 + 1 Status	
		No (n = 4029)	Yes (n = 4029)
Age, median (IQR), y	70 (66-75)	70 (66-75)	70 (66-75)
Gender			
Male	3267 (40.5)	1641 (40.7)	1626 (40.4)
Female	4791 (59.5)	2388 (59.3)	2403 (59.6)
Race and ethnicity			
Asian	54 (0.7)	27 (0.7)	27 (0.7)
Black	310 (3.8)	155 (3.8)	155 (3.8)
Hispanic	44 (0.5)	22 (0.5)	22 (0.5)
White	7584 (94.1)	3793 (94.1)	3791 (94.1)
Other^b^	66 (0.8)	32 (0.8)	34 (0.8)
Smoking history or current			
No	656 (8.1)	320 (7.9)	336 (8.3)
Yes	7402 (91.9)	3709 (92.1)	3693 (91.7)
Clinical T stage			
T1	7091 (88.0)	3550 (88.1)	3541 (87.9)
T2	836 (10.4)	412 (10.2)	424 (10.5)
T3	131 (1.6)	67 (1.7)	64 (1.6)
Surgical type			
Wedge	1310 (16.3)	655 (16.3)	655 (16.3)
Segment	1242 (15.4)	621 (15.4)	621 (15.4)
Lobe	5506 (68.3)	2753 (68.3)	2753 (68.3)
Cardiovascular comorbidity (at least 1)			
No	1419 (17.6)	703 (17.4)	716 (17.8)
Yes	6639 (82.4)	3326 (82.6)	3313 (82.2)
Anatomical tumor location			
RUL	3063 (38.0)	1532 (38.0)	1531 (38.0)
RML	366 (4.5)	183 (4.5)	183 (4.5)
RLL	1446 (17.9)	717 (17.8)	729 (18.1)
LUL	2166 (26.9)	1089 (27.0)	1077 (26.7)
LLL	1017 (12.6)	508 (12.6)	509 (12.6)
Surgical approach			
Open	344 (4.3)	172 (4.3)	172 (4.3)
VATS	3040 (37.7)	1520 (37.7)	1520 (37.7)
Robotic	4674 (58.0)	2337 (58.0)	2337 (58.0)

Abbreviations: LLL, left lower lobe; LUL, left upper lobe; RLL, right lower lobe; RML, right middle lobe; RUL, right upper lobe; VATS, video-assisted thoracic surgery.

^a^ Unless otherwise indicated.

^b^ No further description in the STS Data Dictionary.

**Table 2.** Propensity Score–Adjusted Postoperative Complications and Outcomes

Variable	No. (%) of complications or outcomes^a^			*P* value^b^
	Total (N = 8058)	No (n = 4029)	3 + 1 (n = 4029)	
Postoperative complications				
Postoperative events				
Yes	2396 (29.7)	1208 (30.0)	1188 (29.5)	.33
No	5652 (70.2)	2819 (70.0)	2833 (70.4)	
Patient died in OR	8 (0.1)	2 (0.0)	6 (0.1)	
Atrial arrhythmia				
Yes	566 (7.0)	282 (7.0)	284 (7.1)	.92
No	7482 (93.0)	3745 (93.0)	3737 (92.9)	
Pleural effusion requiring drainage				
Yes	146 (1.8)	63 (1.6)	83 (2.1)	.09
No	7900 (98.2)	3963 (98.4)	3937 (97.9)	
Postoperative pRBCs				
Yes	144 (1.8)	83 (2.1)	61 (1.5)	.07
No	7904 (98.2)	3944 (97.9)	3960 (98.5)	
Air leak				
Yes	931 (11.6)	467 (11.6)	464 (11.5)	.94
No	7117 (88.4)	3560 (88.4)	3557 (88.5)	
Postoperative therapeutic bronchoscopy				
Yes	194 (2.4)	103 (2.6)	91 (2.3)	.39
No	7853 (97.6)	3924 (97.4)	3929 (97.7)	
Pneumonia				
Yes	190 (2.4)	97 (2.4)	93 (2.3)	.78
No	7858 (97.6)	3930 (97.6)	3928 (97.7)	
Respiratory failure				
Yes	123 (1.5)	69 (1.7)	54 (1.3)	.18
No	7925 (98.5)	3958 (98.3)	3967 (98.7)	
Bronchopleural fistula				
Yes	12 (0.1)	3 (0.1)	9 (0.2)	.08
No	8036 (99.9)	4024 (99.9)	4012 (99.8)	
Pulmonary embolism				
Yes	35 (0.4)	14 (0.3)	21 (0.5)	.23
No	8013 (99.6)	4013 (99.7)	4000 (99.5)	
Pneumothorax				
Yes	275 (3.4)	128 (3.2)	147 (3.7)	.24
No	7773 (96.6)	3899 (96.8)	3874 (96.3)	
Ventilatory support >48 h				
Yes	8 (0.1)	4 (0.1)	4 (0.1)	>.99
No	8040 (99.9)	4023 (99.9)	4017 (99.9)	
Ventricular arrhythmia				
Yes	22 (0.3)	12 (0.3)	10 (0.2)	.67
No	8025 (99.7)	4014 (99.7)	4011 (99.8)	
Myocardial infarction				
Yes	15 (0.2)	9 (0.2)	6 (0.1)	.44
No	8033 (99.8)	4018 (99.8)	4015 (99.9)	
Sepsis				
Yes	48 (0.6)	21 (0.5)	27 (0.7)	.38
No	8000 (99.4)	4006 (99.5)	3994 (99.3)	
Chyle leak				
Yes	62 (0.8)	27 (0.7)	35 (0.9)	.30
No	7985 (99.2)	4000 (99.3)	3985 (99.1)	
New-onset recurrent laryngeal nerve paresis				
Yes	25 (0.3)	16 (0.4)	9 (0.2)	.16
No	8023 (99.7)	4011 (99.6)	4012 (99.8)	
Discharged with chest tube				
Yes	744 (9.2)	378 (9.4)	366 (9.1)	.01
No	7276 (90.3)	3623 (89.9)	3653 (90.7)	
Unknown	38 (0.5)	28 (0.7)	10 (0.2)	
Clinical outcomes				
Length of stay, d				
Mean (SD)	4.4 (11.5)	4.7 (14.8)	4.0 (6.6)	<.001
Median (IQR)	3 (2-5)	3 (2-5)	3 (2-5)	
OR duration, median (IQR), min	218 (171-278)	217 (169-280)	218 (173-276)	.41
Readmitted within 30 d				
Yes	579 (7.2)	294 (7.4)	285 (7.1)	.77
No	7131 (89.0)	3558 (89.0)	3573 (89.0)	
Unknown	305 (3.8)	147 (3.7)	158 (3.9)	
30-Day mortality				
Alive	7980 (99.0)	3979 (98.8)	4001 (99.3)	.04
Dead	60 (0.7)	38 (0.9)	22 (0.5)	
Unknown	18 (0.2)	12 (0.3)	6 (0.1)	
Pathologic upstaging				
Pathologic upstaging				
No	7198 (89.3)	3614 (89.7)	3584 (89.0)	.45
Yes	845 (10.5)	409 (10.2)	436 (10.8)	
Unknown	15 (0.2)	6 (0.1)	9 (0.2)	
Pathologic upstaging, open				
No	293 (85.2)	153 (89.0)	140 (81.4)	.11
Yes	50 (14.5)	19 (11.0)	31 (18.0)	
Unknown	1 (0.3)	0	1 (0.6)	
Pathologic upstaging, laparoscopic				
No	2728 (89.7)	1370 (90.1)	1358 (89.3)	.77
Yes	306 (10.1)	147 (9.7)	159 (10.5)	
Unknown	6 (0.2)	3 (0.2)	3 (0.2)	
Pathologic upstaging, robotic				
No	4177 (89.4)	2091 (89.5)	2086 (89.3)	.77
Yes	489 (10.5)	243 (10.4)	246 (10.5)	
Unknown	8 (0.2)	3 (0.1)	5 (0.2)	

Abbreviations: OR, operating room; pRBCs, packed red blood cells.

^a^ Unless otherwise indicated.

^b^ *P* < .002 was deemed significant based on Bonferroni correction.

## Discussion

In this study, we used a national database to evaluate postoperative complication rates in patients with early-stage NSCLC who satisfy 3 + 1 LNS. In our unadjusted cohort, patients who satisfied the 3 + 1 criteria had a larger association with pathologic upstaging and longer operative duration. However, this was no longer seen in our adjusted cohort, indicating that this was likely related to confounding clinical characteristics. For example, univariate analysis showed that patients who underwent 3 + 1 LNS had significantly higher rates of pathological upstaging overall and when disaggregated by surgical approach, likely due to receipt of a more oncologically sound, anatomical resection. This difference was no longer seen in the matched cohort, likely due to balancing of important covariates, such as extent of resection. In contrast, mean LOS was statistically different in the postmatch cohort, with patients undergoing 3 + 1 LNS having a decreased LOS (4.0 vs 4.7 days). However, given the large SDs, this is likely not an accurate measure of LOS in this patient population. Median LOS for both groups were identical. Overall, there were no clinically significant differences in postoperative complications between patients who underwent 3 + 1 LNS and those who did not in the adjusted cohort.

Two-thirds of the patients in our cohort underwent guideline-recommended LNS. In a 2022 study, Heiden et al^5^ compared survival outcomes among 9749 patients at the Veterans Health Administration with clinical stage I NSCLC who underwent resection. Patients were classified based on whether they underwent dissection of at least 10 hilar or mediastinal LNs or at least 3 mediastinal nodes and 1 hilar node. The authors found that 26.3% of patients satisfied the 3 + 1 rule, with adherence increasing over time. Our study shows an increased adherence since the Heiden et al^5^ study, suggesting increased adherence over 2 years. We anticipate that receipt of guideline-concordant lymphadenectomy will continue to improve with increased awareness over time. Further research is necessary to identify patient and surgeon-specific prognostic factors that increase adherence.

Current studies comparing MLND vs MLNS largely evaluate long-term survival outcomes. In a meta-analysis by Hughes et al^10^ reviewing 14 high-quality publications, the authors concluded that there was minimal difference in survival between MLND and MLNS in stage I tumors, without increased accuracy in staging with MLND. Heiden et al^5^ found improved recurrence-free survival in patients who satisfied the 3 + 1 criteria but no improvement in overall survival between groups. The study by Heiden et al^5^ underscores a substantial gap in the current literature; the ACS-CoC’s station-based sampling strategy is based on the theory that station location is of more importance than the number of LNs because the existing literature shows significant variability between number of LNs sampled and long-term survival. However, additional studies need to be conducted to examine whether there is benefit in long-term oncologic and survival outcomes with adherence to 3 + 1 LNS because current evidence is limited. However, Heiden et al^5^ do not examine postoperative complications as described in our study, which found 3 + 1 LNS to be a safe sampling method with no increased association with postoperative complications.

In the ACOSOG Z0030 trial,^3^ patients with an ECOG score less than 3 and a tissue diagnosis of clinically resectable T1 or T2, N0 or nonhilar N1, M0 NSCLC underwent LNS of station 2R, 4R, 7, and 10R for right-sided tumors or 5, 6, 7, and 10L for left-sided tumors. For patients randomized to MLNS, no additional LNs were removed. The study showed no difference in overall survival, rate of local or regional recurrence, and 5-year disease-free survival in early-stage NSCLC between 3 + 1 LNS and MLND.^3^ The studied postoperative complications included atrial arrythmia, chest tube for greater than 7 days, air leak for greater than 7 days, atelectasis, respiratory failure, pneumonia, hemorrhage requiring transfusion, hemorrhage requiring reoperation, myocardial infarction, empyema, adult respiratory distress syndrome, chylothorax, recurrent nerve injury, and bronchopleural fistula. There were no statistically significant differences in operative morbidity or mortality between groups. Although recurrent nerve injury and chylothorax rates among the MLND group were more than twice those of the MLNS sampling group, the ACOSOG Z0030 trial^3^ was underpowered to detect a difference in these particular outcomes due to their small sample size. In comparison, our matched cohort showed no difference in postoperative chylothorax or recurrent nerve injury between patients who underwent 3 + 1 LNS and those who did not. Although patients theoretically underwent 3 + 1 LNS based on the protocol, it was completed before randomization and curative intent surgery via mediastinoscopy, thoracotomy, or video-assisted thoracic surgery. Additionally, the comparative groups were MLNS vs MLND compared with our study, which directly compares patients who underwent 3 + 1 LNS vs those who did not. In a 2014 meta-analysis of RCTs, the authors found no significant difference in the rates of arrhythmias, prolonged air leak, and pneumonia between MLND vs MLNS groups.^9^ However, there was heterogeneity in how MLNS was conducted among RCTs; therefore, it cannot be generalized to the 3 + 1 rule.^9^ The meta-analysis was also conducted prior to the recent adoption of the 3 + 1 criteria by NCCN and ACS-CoC.^8^ Although there are studies that describe postoperative complications in patients who undergo LNS, there are no studies that assess short-term morbidity specifically in the 3 + 1 LNS group.

In a prospective institutional database study by Resio et al,^11^ more than 9000 patients with clinical stage I to III lung cancer who underwent pulmonary resections were examined to determine the association between 3 + 1 LNS and clinical outcomes. The authors found 3 + 1 LNS was associated with increased upstaging and in-hospital complications, with no difference in recurrence or mortality in stage I or III NSCLC. Specifically, Clavien-Dindo grade 3 or greater complications, chylothorax, recurrent nerve injury, and bleeding complications were more frequent in the 3 + 1 group. These differences in complications may be affected by the changing landscape of minimally invasive thoracic surgery during the past 2 decades, specifically the rise of robotic-assisted surgery. Similarly, Rocco et al^12^ conducted a retrospective study of 654 patients with clinical stage IA adenocarcinoma and squamous cell carcinoma who underwent resection at a single center. The authors found a higher rate of guideline-concordant LNS in patients who underwent open vs minimally invasive resections. However, minimally invasive resections were not further disaggregated into video-assisted thoracic surgery and robotic-assisted surgery; given the study period of 2005 to 2014, this likely does not capture the increased visualization and dexterity that robotic-assisted LNS provides. In contrast, our study showed a higher rate of guideline-concordant LNS in robotic-assisted resections compared with open resection and video-assisted thoracic surgery. With regard to postoperative complications, the authors found a higher rate of prolonged air leak, a nonstatistically significant rate of chylothorax, longer duration of chest tube drainage, and longer LOS in the 3 + 1 group.^12^ In comparison, our study included patients with clinical T1 to T3 NSCLC not limited by histologic subtype. We did not find statistical differences in the rate of air leak or chylothorax or a clinically significant difference in LOS between the matched cohorts. These differences may be due to both studies^11,12^ querying patient populations before the implementation of the new standards by the ACOG-CoC at single, dedicated cancer center. In contrast, our study includes a larger cohort of patients of 1000 participating surgeons after the ACS-CoC guidelines were formalized, likely capturing a more generalizable patient population and more up-to-date surgeon practices.

### Limitations

As an observational study, these findings should be interpreted in the context of several limitations. Although propensity score matching was used to balance baseline covariates, residual confounding from unmeasured variables may remain. Furthermore, we used a retrospective database, which may introduce selection bias. The STS GTSD is a voluntary database in North America, containing more than 900 participating surgeons, consisting mainly of high-quality centers, which may not capture all patients with early-stage NSCLC who underwent resection with LNS. Participation is not universal and may be skewed toward higher-volume and academic centers. As such, the results of this study may only be generalizable to patients served by surgeons who voluntarily participate in the STS GTSD.^11^

## Conclusions

This study found that the 3 + 1 rule was not associated with increased postoperative complications in a matched cohort of patients with early-stage NSCLC who underwent lung resection. This study’s results support the use of the 3 + 1 rule in terms of overall safety profile for surgeons who elect to use station-based LNS, in concordance with current recommendations from the NCCN and ACS-CoC. Further research is needed to evaluate its effects on long-term survival and local and regional recurrence after implementation of the current recommendations.

## Notes

Supplement 1. **eTable 1. **Overall Demographics – 3+1 Lymph Node Dissection vs. Not **eTable 2. **Unadjusted Post-Operative Complications and Outcomes – 3+1 Lymph Node Dissection vs. Not

Supplement 2.
Data Sharing Statement
