# Supplementary material for: Lymph Node Dissection and Postoperative Complications After Lung Cancer Resection
Source: JAMA Netw Open. 2026 Jun 1;9(6):e2615894. doi: 10.1001/jamanetworkopen.2026.15894 (PMC13227313; doi:10.1001/jamanetworkopen.2026.15894)
Supplement: Supplement 1. — eTable 1. Overall Demographics – 3+1 Lymph Node Dissection vs. Not eTable 2. Unadjusted Post-Operative Complications and Outcomes – 3+1 Lymph Node Dissection vs. Not [file jamanetwopen-e2615894-s001.pdf]

Supplemental Online Content

Madeka I, Noueihed K, Woodroof J, et al. Lymph node dissection and postoperative complications after lung cancer resection. *JAMA Netw Open*. 2026;9(6):e2615894. doi:10.1001/jamanetworkopen.2026.15894

eTable 1. Overall Demographics – 3+1 Lymph Node Dissection vs. Not

eTable 2. Unadjusted Post-Operative Complications and Outcomes – 3+1 Lymph Node Dissection vs. Not

This supplemental material has been provided by the authors to give readers additional information about their work.

© 2026 Madeka I et al. *JAMA Network Open*.

eTable 1. Overall Demographics – 3+1 Lymph Node Dissection vs. Not

Variable	Total (n=28,439), N (%)	No (n=9,500), N (%)	3+1 (n=18,939), N (%)
Age*	69 (64-75)	69 (64-75)	69 (63-75)
Gender			
Male	11,683 (41.1)	3,998 (42.1)	7,685 (40.6)
Female	16,752 (58.9%)	5,500 (57.9)	11,252 (59.4)
Race / Ethnicity			
Asian	1,254 (4.4)	341 (3.6)	913 (4.8)
Black	2,412 (8.5)	754 (7.9)	1,658 (8.8)
Hispanic	1,048 (3.7)	357 (3.8)	691 (3.6)
White	22,784 (80.1)	7,733 (81.4)	15,051 (79.5)
Other	941 (3.3)	315 (3.3)	626 (3.3)
Pulmonary Function Tests			
Performed	27,230 (95.8)	9,020 (94.9)	18,210 (96.2)
Not Performed	1,208 (4.2)	480 (5.1)	728 (3.8)
Smoking History or Current			
No	5,212 (18.3)	1,717 (18.1)	3,495 (18.5)
Yes	23,227 (81.7)	7,783 (81.9)	15,444 (81.5)
Clinical T Stage			
T1	22,371 (78.7)	7,766 (81.7)	14,605 (77.1)
T2	4,666 (16.4)	1,344 (14.1)	3,322 (17.5)
T3	1,402 (4.9)	390 (4.1)	1,012 (5.3)
Surgical Type			
Wedge	4,496 (15.8)	2,534 (26.7)	1,962 (10.4)
Segment	4,667 (16.4)	1,547 (16.3)	3,120 (16.5)
Lobe	19,063 (67.0)	5,356 (56.4)	13,707 (72.4)
Pneumonectomy	213 (0.7)	63 (0.7)	150 (0.8)
Pathologically Upstaged			
No	24,938 (87.7)	8,546 (90.0)	16,392 (86.6)
Yes	3,442 (12.1)	922 (9.7)	2,520 (13.3)
Unknown	59 (0.2)	32 (0.3)	27 (0.1)
Cardiovascular Comorbidity (at least one)			
No	7,195 (25.3)	2,350 (24.7)	4,845 (25.6)
Yes	21,244 (74.7)	7,150 (75.3)	14,094 (74.4)
American Society of Anesthesiologists Risk Scale			
Normal	35 (0.1)	13 (0.1)	22 (0.1)
Mild Syst Disease	3,471 (12.2)	1,127 (11.9)	2,344 (12.4)

© 2026 Madeka I et al. *JAMA Network Open.*

Severe Syst Disease	23,156 (81.4)	7,755 (81.6)	15,401 (81.3)
Life threatening	1,770 (6.2)	604 (6.4)	1,166 (6.2)
Moribund	3 (0.0)	1 (0.0)	2 (0.0)
Brain Dead	4 (0.0)	0 (0.0)	4 (0.0)
ECOG Score			
Fully Active	16,606 (68.6)	5,300 (67.7)	11,306 (69.0)
Restricted	6,629 (27.4)	2,136 (27.3)	4,493 (27.4)
No work activities	856 (3.5)	339 (4.3)	517 (3.2)
Limited self-care	116 (0.5)	52 (0.7)	64 (0.4)
Completely Disabled	10 (0.0)	4 (0.1)	6 (0.0)
Anatomical Tumor Location			
RUL	9,392 (33.7)	3,151 (33.8)	6,241 (33.7)
RML	1,606 (5.8)	589 (6.3)	1,017 (5.5)
RLL	5,331 (19.1)	1,828 (19.6)	3,503 (18.9)
LUL	7,257 (26.1)	2,365 (25.3)	4,892 (26.4)
LLL	4,261 (15.3)	1,399 (15.0)	2,862 (15.5)
Surgical Approach			
Open	2,326 (8.2)	945 (10.0)	1,381 (7.3)
VATS	9,682 (34.1)	4,270 (45.0)	5,412 (28.6)
Robotic	16,404 (57.7)	4,270 (45.0)	12,134 (64.1)

*Age presented as median (IQR)

Abbreviations: RUL, Right Upper Lobe; RML, Right Middle Lobe; RLL, Right Lower Lobe; LUL, Left Upper Lobe; LLL, Left Lower Lobe; VATS, Video-Assisted Thoracic Surgery

eTable 2. Unadjusted Post-Operative Complications and Outcomes – 3+1 Lymph Node Dissection vs. Not

**Variable**	**Total (n=28,439), N (%)**	**No (n=9,500), N (%)**	**3+1 (n=18,939), N (%)**
Post-Operative Complications			
Post-Operative Events			
Yes	7,921 (27.9)	2,618 (27.6)	5,303 (28.0)
No	20,491 (72.1)	6,872 (72.3)	13,619 (71.9)
Pt Died in OR	24 (0.1)	9 (0.1)	15 (0.1)
Atrial Arrhythmia			
Yes	1,873 (6.6)	604 (6.4)	1,269 (6.7)
No	26,539 (93.4)	8,886 (93.6)	17,653 (93.3)
Pleural Effusion Requiring Drainage			
Yes	556 (2.0)	161 (1.7)	395 (2.1)

© 2026 Madeka I et al. *JAMA Network Open.*

No	27,853 (98.0)	9,328 (98.3)	18,525 (97.9)
Post-Operative pRBCs			
Yes	547 (1.9)	208 (2.2)	339 (1.8)
No	27,865 (98.1)	9,282 (97.8)	18,583 (98.2)
Air Leak			
Yes	2,790 (9.8)	955 (10.1)	1,835 (9.7)
No	25,621 (90.2)	8,535 (89.9)	17,086 (90.3)
Post-Operative Therapeutic Bronchoscopy			
Yes	629 (2.2)	203 (2.1)	426 (2.3)
No	27,782 (97.8)	9,287 (97.9)	18,495 (97.7)
Pneumonia			
Yes	666 (2.3)	212 (2.2)	454 (2.4)
No	27,746 (97.7)	9,278 (97.8)	18,468 (97.6)
Respiratory Failure			
Yes	410 (1.4)	134 (1.4)	276 (1.5)
No	28,002 (98.6)	9,356 (98.6)	18,646 (98.5)
Bronchopleural Fistula			
Yes	54 (0.2)	13 (0.1)	41 (0.2)
No	28,358 (99.8)	9,477 (99.9)	18,881 (99.8)
Pulmonary Embolism			
Yes	139 (0.5)	39 (0.4)	100 (0.5)
No	28,272 (99.5)	9,451 (99.6)	18,821 (99.5)
Pneumothorax			
Yes	856 (3.0)	271 (2.9)	585 (3.1)
No	27,555 (97.0)	9,219 (97.1)	18,336 (96.9)
Ventilatory Support > 48 hrs			
Yes	26 (0.1)	12 (0.1)	14 (0.1)
No	28,386 (99.9)	9,478 (99.9)	18,908 (99.9)
Ventricular Arrhythmia			
Yes	91 (0.3)	38 (0.4)	53 (0.3)
No	28,320 (99.7)	9,451 (99.6)	18,869 (99.7)
Myocardial Infarction			
Yes	59 (0.2)	19 (0.2)	40 (0.2)
No	28,353 (99.8)	9,471 (99.8)	18,882 (99.8)
Sepsis			
Yes	143 (0.5)	41 (0.4)	102 (0.5)
No	28,269 (99.5)	9,449 (99.6)	18,820 (99.5)
Chyle Leak			
Yes	224 (0.8)	57 (0.6)	167 (0.9)
No	28,183 (99.2)	9,433 (99.4)	18,750 (99.1)

© 2026 Madeka I et al. *JAMA Network Open*.

New onset recurrent laryngeal nerve paresis			
Yes	77 (0.3)	28 (0.3)	49 (0.3)
No	28,331 (99.7)	9,460 (99.7)	18,871 (99.7)
Discharged with Chest Tube			
Yes	2,264 (8.0)	793 (8.3)	1,471 (7.8)
No	26,033 (91.5)	8,654 (91.1)	17,379 (91.8)
Unknown	142 (0.5)	53 (0.6)	89 (0.5)
Clinical Outcomes			
Length of Stay (Days)			
Mean (SD)	4.1 (10)	4.3 (11.4)	4.1 (9.2)
Median (IQR [Interquartile Range])	3 (2-5)	3 (2-5)	3 (2-5)
OR Duration (Minutes), Median (IQR)*	219 (172-278)	210 (161-273)	224 (178-281)
Readmitted within 30 Days			
Yes	1,971 (7.0)	649 (6.9)	1,322 (7.0)
No	25,209 (89.2)	8,437 (89.4)	16,772 (89.1)
Unknown	1,089 (3.9)	353 (3.7)	736 (3.9)
30 Day Mortality			
Alive	28,179 (99.1)	9,410 (99.1)	18,769 (99.1)
Dead	207 (0.7)	65 (0.7)	142 (0.7)
Unknown	46 (0.2)	22 (0.2)	24 (0.1)
Pathologic Upstaging			
Pathologic Upstaging*			
No	24,938 (87.7)	8,546 (90.0)	16,392 (86.6)
Yes	3,442 (12.1)	922 (9.7)	2,520 (13.3)
Unknown	59 (0.2)	32 (0.3)	27 (0.1)
Pathologic Upstaging – Open*			
No	1,871 (80.4)	798 (84.4)	1,073 (77.7)
Yes	445 (19.1)	140 (14.8)	305 (22.1)
Unknown	10 (0.4)	7 (0.7)	3 (0.2)
Pathologic Upstaging – Laparoscopic*			
No	8,653 (89.4)	3,900 (91.3)	4,753 (87.8)
Yes	1,005 (10.4)	353 (8.3)	652 (12.0)
Unknown	24 (0.2)	17 (0.4)	7 (0.1)
Pathologic Upstaging – Robotic*			
No	14,398 (87.8)	3,836 (89.8)	10,562 (87.0)
Yes	1,981 (12.1)	426 (10.0)	1,555 (12.8)
Unknown	25 (0.2)	8 (0.2)	17 (0.1)

© 2026 Madeka I et al. *JAMA Network Open*.

*Outcomes deemed significant. P-value of < 0.002 was deemed significant based on Bonferroni correction.

© 2026 Madeka I et al. *JAMA Network Open*.
