# Supplementary material for: Lymph Node Dissection and Postoperative Complications After Lung Cancer Resection
Source: JAMA Netw Open. 2026 Jun 1;9(6):e2615894. doi: 10.1001/jamanetworkopen.2026.15894 (PMC13227313; doi:10.1001/jamanetworkopen.2026.15894)
Supplement: Supplement 2. — Data Sharing Statement [file jamanetwopen-e2615894-s002.pdf]

Data Sharing Statement

Madeka. Lymph Node Dissection and Postoperative Complications After Lung Cancer Resection. *JAMA Netw Open*. Published June 01, 2026.
doi:10.1001/jamanetworkopen.2026.15894

Data
Data available: No

Additional Information
Explanation for why data not available: This data is from the Society of Thoracic Surgeons General Thoracic Surgery Database; any data sharing is per the STS-GTSD.
